# Implementation of a threefold intervention to improve palliative care for persons experiencing homelessness: a process evaluation using the RE-AIM framework

**DOI:** 10.1186/s12904-022-01083-3

**Published:** 2022-11-04

**Authors:** Hanna T. Klop, Anke J. E. de Veer, Jaap R. G. Gootjes, Marieke Groot, Judith A. C. Rietjens, Bregje D. Onwuteaka-Philipsen

**Affiliations:** 1 Department of Public and Occupational Health, Amsterdam UMC, Vrije Universiteit Amsterdam, Amsterdam Public Health research institute (APH), De Boelelaan 1117, Amsterdam, Netherlands; 2 Netherlands Institute for Health Services Research (Nivel), Otterstraat 18, Utrecht, Netherlands; 3 Hospice Kuria, Valeriusplein 6, Amsterdam, Netherlands; 4 Expertise Centre for Palliative Care, Amsterdam UMC location VUmc, De Boelelaan 1117, Amsterdam, Netherlands; 5 Research Centre Innovations in Care, University of Applied Sciences Rotterdam, Rotterdam, Netherlands; 6 Department of Public Health, Erasmus MC, University Medical Center Rotterdam, Rotterdam, Netherlands

**Keywords:** Palliative care, End of life, Homeless, Intervention, Consultation, Social services, Professionals

## Abstract

**Background:**

Palliative care provision for persons experiencing homelessness is often poor. A threefold consultation service intervention was expected to increase knowledge of palliative care and multidisciplinary collaboration, and improve palliative care for this population. This intervention comprised: 1) consultation of social service professionals by palliative care specialists and vice versa; 2) multidisciplinary meetings with these professionals; and 3) training and education of these professionals. We aimed to evaluate the implementation process and its barriers and facilitators of this service implemented within social services and healthcare organizations in three Dutch regions.

**Methods:**

A process evaluation using structured questionnaires among consultants, semi-structured individual and group interviews among professionals involved, and (research) diaries. Qualitative data were analysed using thematic analysis. The process evaluation was structured using the Reach, Adoption, Implementation and Maintenance dimensions of the RE-AIM framework.

**Results:**

All three regions adopted all three activities of the intervention, with differences between the three regions in the start, timing and frequency. During the 21-month implementation period there were 34 consultations, 22 multidisciplinary meetings and 9 training sessions. The professionals reached were mainly social service professionals. Facilitators for adoption of the service were a perceived need for improving palliative care provision and previous acquaintance with other professionals involved, while professionals’ limited skills in recognizing, discussing and prioritizing palliative care hindered adoption. Implementation was facilitated by a consultant’s expertise in advising professionals and working with persons experiencing homelessness, and hindered by COVID-19 circumstances, staff shortages and lack of knowledge of palliative care in social service facilities. Embedding the service in regular, properly funded meetings was expected to facilitate maintenance, while the limited number of persons involved in this small-scale service was expected to be an obstacle.

**Conclusions:**

A threefold intervention aimed at improving palliative care for persons experiencing homelessness is evaluated as being most usable when tailored to specific regions, with bedside and telephone consultations and a combination of palliative care consultants and teams of social service professionals. It is recommended to further implement this region-tailored intervention with palliative care consultants in the lead, and to raise awareness and to remove fear of palliative care provision.

**Supplementary Information:**

The online version contains supplementary material available at 10.1186/s12904-022-01083-3.

## Background

Persons experiencing homelessness often suffer from varying combinations of problems such as intellectual disabilities [1], a high burden of somatic and psychiatric problems [2], substance use [3], high symptom burden and high rates of early morbidity compared to the general population [4, 5]. In the Netherlands, about 32,000 persons are officially registered as using the Dutch social services system for persons experiencing homelessness [6]. Social services in the Netherlands provide temporary accommodation and professional help, including emergency shelters with a place to sleep and/or to spend the day. Within these social service facilities, medical services can be available [7].

Given the high morbidity and mortality in this group, part of the persons experiencing homelessness will need palliative care [8]. Traditional palliative care services such as hospices, home care or General Practitioners (GPs) often not succeed in reaching persons experiencing homelessness who are seriously ill and could be at the end of life. Appropriate housing, home care, and thus palliative care, as well as respite or hospice facilities who are open for this population are often lacking [9–12]. Palliative care is often provided late or not at all to persons experiencing homelessness. Even if palliative care is provided, the provision to persons experiencing homelessness is characterized by many impediments such as lack of expertise and training in characteristics of this population among professionals in palliative care services, and lack of expertise and training in palliative care. Persons experiencing homelessness typically have chaotic lifestyles and an unpredictable course of illness, with unexpected improvements in their health if they are cared for in social services [13–15]. Examples of impediments in care provision to this population are: a lack of criteria pertaining to when a person experiencing homelessness is eligible for referral to palliative care or hospice care [16]; what is often perceived to be the patronizing and stigmatizing attitude of healthcare staff towards persons experiencing homelessness [17, 18]; a large number of different social service professionals involved in the delivery of daily care [14]; and inflexibility in mainstream healthcare systems in adapting the care to the specific needs of a person experiencing homelessness. Moreover, improving palliative care for this population is complex because of the decentralized organization of social and healthcare services. This results in individual municipalities or regions consequently taking an individual approach and differing from each other in the range and possibilities of services [19]. Also, existing regulations and financing systems hinder improvements to palliative care, as we found in our focus group study of barriers and needs regarding the provision of palliative care to persons experiencing homelessness, which we performed as an exploratory study in preparation of the development of the intervention under study here [19]. In this study, we also found that many disciplines are involved when a person experiencing homelessness at the end of life. Good quality palliative care is focused on improving the quality of life of patients and their families and on prevention and relief of suffering by the early identification and impeccable assessment and treatment of pain and other physical, psychosocial and spiritual problems, [20] as defined by the WHO. However, palliative care is often not provided for persons experiencing homelessness, or only to an insufficient degree. Moreover, the provision of palliative care is complicated by the characteristics of this population [14, 18, 21, 22].

In this paper, ‘persons experiencing homelessness’ are defined as persons without housing, who reside in emergency accommodation or accommodation for persons experiencing homelessness or who reside temporarily at a friend’s or relative’s place, as officially defined by Statistics Netherlands (CBS) [6, 23]. In the Netherlands, these persons often reside in social service facilities that provide daytime or overnight stays or temporary housing. Palliative care for people experiencing homelessness is delivered in various settings, such as in-shelter nursing care, outreach home care, or hospices [19, 24], and by various healthcare professionals. In addition, these settings vary across towns and cities. In our exploratory study we performed in preparation of the development of the intervention, we found that professionals employed in social services, healthcare and palliative care indicated that they would benefit from a reciprocal consultation service in order to foster appropriate and timely palliative care [19]. This study showed that professionals expected added value of an adapted version of a local consultation service, which takes the form of a threefold reciprocal intervention. Furthermore, the explorative study identified three core elements of the intervention that were expected important (1) consultations about persons experiencing homelessness and eligible for palliative care, between social-service providers working in the field of services for this population and palliative-service providers, such as hospices and GPs; (2) multidisciplinary meetings between social-service providers and palliative-care professionals to discuss persons experiencing homelessness who are eligible for palliative care; and (3) training and education on subjects related to palliative care and homelessness. According to the preliminary explorative study, this intervention was expected to work best when developed regionally and tailored to the regional situation [19].

Following this preparatory study, during 21 months, a threefold consultation service consisting of the three core elements was implemented in three regions in Dutch healthcare and social service settings. By implementing this service, we aimed to increase collaboration and knowledge as well as improve the quality and timeliness of palliative care delivered to persons experiencing homelessness.

Duo’s of consultants were formed in each region by seeking a ‘strategic partnerships’ consisting of one consultant in palliative care and one consultant in services for persons experiencing homelessness. This duo of consultants formed the basis for the intervention; the consultants took charge of the practical implementation of consultations, multidisciplinary meetings, training and involvement of other organizations. In order to learn from other regions group meetings with all consultants took place every six months. Consultations were given by palliative care experts from the region (determined regionally in the implementation plans), including palliative care nurse specialists and geriatric nurses.

A design feature of the intervention was the context-sensitive approach and implementation plans in order to fit local needs and to tie in with existing collaboration efforts and/or further develop them. The regions of Amsterdam, Rotterdam and Utrecht participated in this intervention. Part of the context-sensitive design was working strategies written down in detailed implementation plans. These implementation plans concerned details of the organization of consultations; existing initiatives for consultation, collaboration, knowledge exchange, and training; the organization of multidisciplinary meetings and potential for improvement; the organization of training and additional educational requirements; needs barriers and facilitators for all three elements; characteristics specific for each region; and possibilities for future financing and future continuation and embedding of the intervention.

Implementation plans were made in the preparatory phase that lasted from June to September 2019. updated every six months on the initiative of the researcher. After this the plans were implemented. This was just months before the start of the COVID-19 pandemic. Every 6 months the implementation plans were evaluated by the regional teams. Persons eligible for palliative care were persons about who were doubts, concerns or questions, or who deteriorated.

The perceived added value of the intervention was described earlier [19]. Perceived added value was found in all three regions for the collaboration and networks of the professionals involved (connecting disciplines reciprocally and strengthening collaboration), the competences of the professionals involved (competency in palliative care provision and feeling emotionally supported in complex situations), and the quality and timing of palliative care (focus on quality of life and dying, advance care planning, and awareness of death and palliative care).

As this threefold intervention is a new phenomenon, a process evaluation was embedded in the implementation process. It was based on the Reach, Adoption, Implementation and Maintenance dimensions of the RE-AIM framework used to structure these different implementation factors [25].

The study was guided by the following research questions:

- 1. What is the Reach, Adoption, Implementation and Maintenance of a threefold consultation function according to the social service and palliative care professionals involved in the threefold intervention?
- 2. What are the perceived barriers and facilitators during this implementation process?

## Methods

### Design of the process evaluation

The process evaluation was designed to systematically monitor and evaluate the implementation of the threefold consultation service approach in three regions in the Netherlands. The RE-AIM framework was used to underpin and structure the analysis and the manuscript. It is an appropriate framework to evaluate the process and implementation of interventions in practice at both the individual level (e.g. healthcare professionals) and the organizational level (e.g. institution, policy) and ensures having attention for the context in which an intervention is implemented. RE-AIM also provides useful starting points for improvement in the further implementation and future maintenance. Therefore, this manuscript is structured following the RE-AIM dimensions and facilitators and barriers corresponding to these dimensions, reporting on the process evaluation in terms of Reach, Adoption, Implementation and Maintenance [25, 26]. Table 1 shows the operationalization for all dimensions of the RE-AIM framework. As we reported on Effectiveness, operationalized as added value, in another study [27], effectiveness was not part of this process evaluation.

**Table 1:** Operationalization of RE-AIM dimensions and CFIR constructs

RE-AIM dimension	Conceptualization of RE-AIM dimension	Operationalization
Reach	To what extent is the target population is reached by the initiatives?	Extent, type and setting of professionals in social, hostel and palliative care services, and homeless persons who were (or were not) involved in consultation, multidisciplinary meetings and training during the study
Adoption	To what degree are the initiatives adopted or used by organizations and settings?	Extent to which consultation, multidisciplinary meetings and training were adopted and used by organizations, standardization of use, and factors affecting this
Implementation	To what extent have the initiatives been implemented according to plan?	Extent to which the threefold consultation services are implemented according to original plans
Maintenance	To what extent are the initiatives are future-proof?	Extent to which the threefold consultation services are (or are expected to be) used, supported and sustained over time by healthcare professionals and management
**Data sources**	**Detailed description of measurement type**	**Timing**
	**1.** Weekly structured digital diary for the consultants, questions on type and number of activities performed, reason for activity, experiences with activity. The activities were: consultations, multidisciplinary meetings, training (given or received), and project team meetings	Month 3–21
	**2.** Structured questionnaire for advising consultant and requesting consultant after each consultation; nature of care request, patient diagnosis, advice provided or received (broken down into the physical, psychological, social, and spiritual domains of palliative care, plus addiction), consultant’s knowledge, consultation timing, facilitating and impeding factors regarding consultation, consultation quality, concreteness and usefulness of advice, effect on quality of palliative care, and added value of consultations	Month 3–21
	**3.** Structured questionnaire after each multidisciplinary meeting filled out by the consultant involved; questions on professional background of attendees, diagnosis, and details of the patients and domains discussed	Month 3–21
	**4.** Semi-structured group interviews with attendees of multidisciplinary meetings and training activities, guided by a topic list with topics on the process of getting involved in multidisciplinary meetings and training activities, appreciation of collaboration and discussions, topics discussed, added value of meetings, effect on knowledge and competences, effect on timing and quality of palliative care, suggestions for improvement	Month 3–21, after MDM
	**5.** Semi-structured individual and group interviews with managers, guided by a topic list with topics on activities, process, added value, and maintenance of intervention activities	Month 15–17
	**6.** Semi-structured short individual interviews with consultants on current activities, collaboration, implementation and effort required, useful elements, missing aspects, perceived benefits of the three elements, perceived added value for collaboration, competences, quality and timing of palliative care	Month 9–12
	**7.** Implementation diary, filled out weekly by the researcher with observations on the intervention: activities performed, steps taken to accomplish this, and evaluations and difficulties in this process. Observations on implementation: support for this process, strategies	Month 1–21
**CFIR Construct**	**Operationalization**	
Intervention characteristics	The intervention as referred to in the project proposal with consultations, MDMs and training activities aimed at palliative care for this population specifically, characteristics of the intervention (reciprocity, duos, bedside consultations), and the activities and perspectives of the professionals involved in this	
Outer setting	The characteristics of organizations and networks that are involved later on and are complementary to the initial collaboration as described in the work plan	
Inner setting	The characteristics and culture of the organizations that were involved in the project from the start as well as the implementation climate within these organizations	
Characteristics of individuals	Characteristics of individuals involved in the project from the start and later on in the project in consultations, MDMs and training	
Process	Process of preparing, planning and executing the intervention	

The process evaluation started during the preparatory phase for all three regions from June until September 2019, and was followed by an evaluation of 18 months of practice, in which the professionals in the regions worked with the intervention.

### Ethical approval

Written or verbal informed consent was provided by all professionals involved in group and individual interviews prior to the interview. Transcripts were anonymized to ensure the participants’ anonymity. Access to the data was limited to two researchers. On 24 July 2019, the Ethics Review Committee of VU University Medical Center provided a waiver as ethical approval was not needed under Dutch law. Ethical considerations for different data collection methods were the novelty of the intervention and thereby obtaining a broad picture of the process.

### Data collection

This process evaluation consisted of structured questionnaires filled out by (requesting and advising) consultants, semi-structured topic-list-guided interviews in which managers, multidisciplinary meeting (MDM) members and consultants participated, structured diaries kept by consultants and an implementation diary kept by the lead author. The topics covered in each data source are listed in Table 1. Table 1 also shows the timing of the data collection. All RE-AIM dimensions got attention in in each data source. Topic lists used during interviews are shown in Additional file 1: Appendix 1.

### Data analysis

Qualitative data collected in semi-structured individual and group interviews and the implementation diary and structured diaries were analysed following the principles of thematic analysis to identify important themes [28]. First, an open thematic analysis was performed to explore the data. After that, the data were structured using the Reach, Adoption, Implementation and Maintenance dimension of the RE-AIM framework [25]. Using MaxQDA (version 2020), analysis started after conducting the first five interviews. After that, topic lists were adjusted slightly as some topics overlapped. Three researchers (HK, BDO, AJEV) independently coded four transcripts and then discussed themes until agreement was achieved. After that, all other data were coded by one researcher (HK). All data were coded using the RE-AIM dimensions. Subsequently, we searched openly for themes concerning barriers and facilitators within each of the RE-AIM dimensions. Then barriers and facilitators within the RE-AIM dimensions were further categorized using predefined constructs of the Consolidated Framework for Implementation Research (CFIR) [29], as operationalized in Table 1. All themes, constructs, quotes and categorizations were discussed in the research team. Answers to open questions were categorized by one researcher (HK) and checked by a second researcher (BDO). Descriptive analyses took place for the quantitative data using SPSS 26.0.

## Results

Results are based on data from 216 structured weekly digital diaries, 34 questionnaires filled out by consultants and 14 questionnaires filled out by requesting consultants, 22 questionnaires completed by MDM attendees, eight semi-structured individual interviews with managers in organizations involved in the intervention, two semi-structured group interviews on MDMs and two semi-structured group interviews on training, five interviews with consultants, and the researcher’s implementation diary. A total of 22 persons were discussed in the consultations and 32 persons in the MDMs, resulting in a total of 54 persons.

### Reach of the intervention

Data sources for Reach were the weekly digital diaries, questionnaires filled out by consultants, requesting consultants and MDM attendees. Barriers and facilitators were identified from the individual and group interviews. Regarding persons experiencing homelessness at the end of life, the intervention mainly reached seriously ill residents of social service facilities (long-term or short-term care); no persons living on the street were reached. Persons discussed in consultations and MDMs were often older persons (42% aged 61 and over) where a need for palliative care was recognized. The professionals reached by the intervention were mainly the colleagues of consultants and/or the team to which the consultant was connected. Prior to the implementation period, the professionals involved estimated the number of social service professionals and palliative care professionals who potentially could be reached by the intervention. Table 2 shows an estimated 400 professionals could potentially be reached by the intervention, and 166 professionals were ultimately reached by the intervention in practice.

**Table 2:** Reach in terms of numbers and professionals

	**n Actual organizations involved**	**n of Professionals who could potentially be reached (estimated)**^**b**^	**n Palliative care and healthcare professionals involved**	**n Social service professionals involved**
Region 1	3	115	19	40
Region 2	4	110	18	19
Region 3	2^a^	175	33	47
Total N	9	400	60	106

^a^This involved two different branches of one large organization: a social services branch and a general district nursing branch that had not previously worked together

^b^Estimated numbers are based on a survey before the start of the intervention among the participating consultants

Table 3 shows intervention activities per region. There were a total number of 34 consultations, 22 MDMs and 9 training sessions. The participants were mainly social service professionals and nurses employed in social service provision. Home-care professionals, hospice nurses, practice nurses and general practitioners were also involved to a lesser extent. During the implementation period, some external professionals other than the initial participants were reached by the intervention, such as professionals working in other social service organizations on the possible extension of the intervention, and professionals in hospitals such as anaesthesiologists and surgeons.

**Table 3:** Number of consultations, MDMs and training sessions per region

	**n Consultations**	**n MDM’s**	**n Training/education**
Region 1	5	11	3
Region 2	5	7	3
Region 3	24	4	3
Total N	34	22	9

The main facilitators in reaching professionals were intervention characteristics and the characteristics of individuals, respectively persons experiencing homelessness already known to the social services involved in the intervention and having an enthusiastic and proactive consultant (Table 4). Reported barriers to reaching persons experiencing homelessness at the end of life were mainly concerned characteristics of the intervention. Telephone consultations and consultations during MDMs were considered to be barriers, as the consultant was not personally able to assess the symptoms and the person itself. Another barrier in reaching this population was that not all seriously ill persons experiencing homelessness for whom a palliative care approach could be beneficial resided in social services or on a nursing ward. Barriers to reaching more social service professionals in the intervention concerned difficulties in getting other social service professionals involved because new contacts had to be made and developed. Regarding the process, COVID-19 restrictions formed a barrier to reaching persons experiencing homelessness and to professionals providing palliative care to them because of visiting restrictions and the high workload of social service professionals due to the COVID-19 pandemic.

**Table 4:** Overview of facilitators and barriers in the RE-AIM Reach dimension

CFIR domain^a^	Facilitators	Barriers
Intervention characteristics	• Persons experiencing homelessness are known to the social services so that the intervention can be potentially beneficial for them	• Telephone consultations and indirect consultations may have hindered the assessment of symptoms and the patients themselves by the consultant • Intervention is aimed at social services, while some persons experiencing homelessness do not reside within these services • Starting the intervention requires time and preparatory work, making it hard to involve new parties as well
Outer setting		• Expanding and reaching additional social service professionals with the intervention is hard to accomplish
Characteristics of individuals	• An enthusiastic and proactive consultant helps in reaching out to social service professionals and establishing intervention activities	
Process		• COVID-19 visiting restrictions and the heavy workload may have hindered efforts to reach persons experiencing homelessness and professionals providing palliative care to them

^**a**^For Reach, we did not find factors for the inner setting

### Adoption of the intervention

**Fig. 1:**
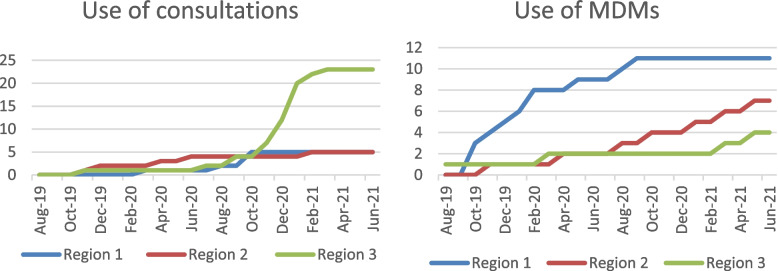
Cumulative numbers of intervention activities in the three regions

Data sources for Adoption were the weekly digital diaries filled out by the consultants. Barriers and facilitators were identified from the individual and group interviews. All three intervention activities —consultations, multidisciplinary meetings and training — were adopted in all three regions. However, the start, timing and frequency of these activities differed greatly from region to region. Figure 1 shows when and how often a region used each of the three activities. The course of activities shows that regular use of the activities required time and effort in preliminary work. The activities were mainly taken up by organizations involved from the start, and occasionally spread to new organizations during the implementation period. Activity growth was mostly gradual. However, in region 3 there was a sudden big increase in consultations due to the regular planned visits of the consultant to the linked social service deployment of professionals. All regions provided three training sessions during the intervention period at a similar pace.

Facilitators in adopting all or parts of the intervention were mainly found in characteristics of individuals, specifically in having a committed, medically skilled and enthusiastic consultant in palliative care and familiar with persons experiencing homelessness (Table 5, illustrated with quotes in Additional file 2: Appendix 2). The intervention is more likely to be adopted if there is a palliative care consultant who is approachable and proactive in offering consultations, training sessions or participation in MDMs. Awareness among social service professionals of shortcomings in palliative care skills makes them open to reflecting and learning, which facilitates adoption of the intervention. Trust between the consultants providing and requesting assistance encourages collaboration and adoption. Intervention characteristics that facilitated adoption were having professionals who perceived a need for palliative care support and saw it as a priority, tools in palliative care for social service professionals, and an intervention tailored to local collaborations and working structures. In line with this, in the outer setting, having pre-existing regular meetings in the networks of the professionals who were involved facilitated the adoption of intervention activities. Facilitating factors identified in the inner setting were the intervention being compatible with current working structures in the organization and a shared vision among collaborating professionals on good healthcare and equal and reciprocal collaboration.

**Table 5:** Overview of all reported facilitators and barriers of the RE-AIM Adoption dimension

CFIR domain	Facilitators	Barriers
Intervention characteristics	• Professionals perceive a need and priority for additional support which makes them feel the need for collaboration (QA1.1)^a^ • Provision of palliative care tools helps consultants concretize palliative care in intervention activities (QA1.2) • An intervention tailored to local collaborations and structures facilitates easy adoption of intervention (QA1.3)	• Benefit of consultations not recognized by all the professionals involved as not everyone feels the need to engage the consultant in bedside consultations (QA1.4) • Concerns about the relative advantage of the intervention as palliative care concerns a small population and narrow topic (QA1.5)
Outer setting	• Pre-existing regular meetings in professionals’ network means that professionals who already have a network among other organizations can easily adopt the intervention (QA1.6)	• Unclear policies on responsibilities. due to too many external parties involved in care for homeless people. may hinder adoption of the intervention (QA1.7)
Inner setting	• Intervention is compatible within existing workflow due to a clear route of palliative care (and responsibilities) within the organization participating in the intervention (QA1.8) • Shared vision on good healthcare among colleagues within the participating organization makes collaboration easier (QA1.9) • Shared views of involved professionals regarding equal and reciprocal cooperation (QA1.10)	• Norms and values within the organization for social service provision are often focused on social care, while somatic care gets less attention (QA1.11) • Limited skills in recognizing, discussing and providing palliative care within social services hinder adequate care at the end of life (QA1.12) • Many staff changes and insecure future prospects for organization make organizational commitment hard (QA1.13) • Limited support and engagement of management makes consultants feel they are getting little support (QA1.14)
Characteristics of individuals	• Commitment and enthusiasm of the professionals involved regarding the intervention and palliative care (QA1.15) • Medical skills and knowledge of individual consultant makes professionals feel the consultant is competent (QA1.16) • An approachable consultant without their own agenda helps the professionals to adapt the intervention according to their needs (QA1.17) • Adoption is facilitated by consultants who proactively initiate consultations, organize training or participate in multidisciplinary meetings, which makes it easier for professionals to adapt to the involvement of the consultant (QA1.18) • A consultant who is familiar with the homeless population makes the intervention easier to adopt for social professionals (QA1.19) • Open mindset and attitude of professionals helps make them more motivated for palliative care (QA1.20) • Awareness of skill shortcomings makes individuals open to reflecting and learning (QA1.21) • Individuals who trust each other cooperate better in using the intervention (QA1.22)	• Low self-efficacy in palliative care skills may hinder identification of palliative care and use of the intervention (QA1.23) • Professionals’ differences in their views when a consultation is requested could hinder use of the intervention (QA1.24)
Process		• Issues with converting the work plan and intentions into actions impedes planning and engaging appropriate individuals (QA1.25) • Unclear implementation route for intervention within organization (QA1.26)

^a^QA refers to quotes on Adoption by facilitating and hindering factors, shown in Additional file 2: Appendix 2

Barriers in adopting the intervention were predominantly found in the CFIR domain of the inner setting. Adoption of the intervention was hindered by norms and values within social services focusing on social care with a focus on recovery, thereby underexposing somatic (palliative) care. In addition, the limited skills of social service professionals in recognizing, discussing and prioritizing palliative care could hinder adoption of the intervention. Staff changes, insecure future prospects for some departments of the social services in question, such as uncertain prospects for nursing beds, and a lack of apparent engagement among managers were also perceived as hindering adoption. With regard to hindering intervention characteristics, the relative advantage of the threefold intervention for such a small population was sometimes questioned.

### Implementation of the intervention

Data sources for Implementation were the questionnaires filled out by consultants, requesting consultants and MDM attendees. Barriers and facilitators were identified from the individual and group interviews. The ‘consultation’ element of the threefold intervention was partly implemented according to plan. Initially, bedside consultations were planned with fixed duos of consultants who consulted each other reciprocally. In practice, 59% of the consultations were held at the bedside and two of the three regions had no fixed duo of consultants, but rather one palliative care consultant and a requesting team of social service professionals. The ‘multidisciplinary meetings’ element was implemented according to plan. The ‘training’ element was implemented according to plan. However, few training sessions were given even though there was a perceived shortage of knowledge and skills. The COVID-19 pandemic played a role in this. Regarding the reciprocity of the duos as originally intended, there was a particular need among professionals in social services for advice and knowledge from the professionals in a palliative setting, because they felt that persons experiencing homelessness were dying more in social services nowadays, with fewer transfers to hospitals or hospices.

Facilitators in the implementation of the intervention were most often mentioned in intervention characteristics and characteristics of individuals (Table 6). Intervention characteristics facilitating implementation were frequent physical consultations, meetings and training sessions, consultants’ structured questioning and working method, and discussing patient cases in training. The fact that the three intervention activities complement one another was also perceived as a facilitator. With regard to characteristics of individuals consultant duo’s who get on well together, colleagues who share tasks in organizing intervention activities, and a strong relationship between social service professionals and persons experiencing homelessness were facilitators. Lastly, in the outer setting, familiarity with other professionals originating from other regular meetings helps in implementing the intervention, as do clear financial structures and regulations regarding palliative care indication for persons experiencing homelessness and financing this care.

**Table 6:** Overview of all reported facilitators and barriers of the RE-AIM Implementation dimension

CFIR domain	Facilitators	Barriers
Intervention characteristics	• Frequent physical meetings (consultations, MDMs, training) normalize collaboration between palliative care professionals and social service professionals (QI1.1)^a^ • Consultants’ structured questioning helps in implementing consultations (QI1.2) • The three intervention activities complement each other, making implementation of the intervention easier (QI1.3) • Discussing patient cases in training helps participants to see the advantage of training (QI1.4) • Making notes of conversations and appointments in patient files contributes to clear agreements (QI1.5)	• Unclear role of consultant increases the perceived complexity of the intervention (QI1.6) • Making limited use of bedside consultation possibilities prevents the consultant from making their own assessments as intended in the intervention (QI1.7) • Still no perceived necessity for MDMs in which palliative care is embedded (QI1.8) • Discussion of patients in MDMs is too short/limited, which hampers implementation of advice in MDM (QI1.9) • Limited time for patient discussion in training hampers implementation of knowledge and skills in training (QI1.10)
Outer setting	• Familiarity with other professionals (not participating in the intervention) through pre-existing regular meetings helps in implementing the intervention (QI1.12) • A policy of clear incentives and regulations regarding palliative care indication and associated (existing) funding helps in implementing the intervention (QI1.13)	• A follow-up consultation was not always possible (QI1.11)
Inner setting	• Available time of consultant supports adaptability of the intervention (QI1.14)	• Staff shortages hinder implementation of the intervention (QI1.15) • Many unexpected events and therefore ad hoc activities within social service organizations distracts from focusing on implementing the intervention (QI1.16) • Implementation can be difficult when social service professionals do not know the consultant yet (QI1.17) • Lack of knowledge on how to recognize palliative care needs frequently led to consultations that were too late in the illness trajectory (QI1.18)
Characteristics of individuals	• Consultant is experienced in advising professionals about this patient population, which helps in introducing implementation activities (QI1.19) • Predictability of the consultants’ presence helps to normalize implementation of intervention activities (QI1.20) • Sharing tasks with colleague helps familiarization with implementing the intervention (QI1.21) • Getting on as consultants helps in implementing consultations (QI1.22) • A personal bond between social service provider and patient helps in ensuring proper use of the intervention (QI1.23) • Professionals perceiving the consultant as highly competent (QI1.24)	• Resistance and fear of end of life and death hinders implementation (QI1.25)
Process		• COVID-19 restrictions and the scaling down of healthcare made planning and implementing intervention activities more difficult (QI1.25)

^a^QI refers to quotes on Implementation by facilitating and hindering factor, shown in Additional file 2: Appendix 2

Barriers in the implementation of the intervention were mainly perceived in intervention characteristics and inner settings of the organizations involved. Barriers in intervention characteristics include a lack of clarity about the role of the consultant in the intervention and consultants not feeling able to assess the situation themselves due to the lack of bedside consultations. No perceived necessity among social service professionals for embedding persons eligible for palliative care in MDMs, and limited time for discussing persons in MDMs and training were also perceived as hampering the implementation of the intervention. In the inner setting of the organizations, staff shortages in social services, unexpected situations and ad hoc activities in the day-to-day business of social services, and late consultation requests sometimes hindered implementation of the intervention. Lastly, regarding the process of implementation, COVID-19 had an effect that could of course not have been predicted. Implementing the threefold intervention was probably particularly difficult due to COVID-19 restrictions and the scaling down of healthcare during the intervention. This might have been an obstacle to planning and using of the intervention.

### Maintenance of the intervention

Data sources for Maintenance were the individual and group interviews. Professionals of all regions expected to continue with the use of one or more activities of the intervention in the future, although the three regions differed in the expected continuation of facets of the threefold intervention. Region 1 preferred MDMs as the activity that was most commonly used, while Region 2 preferred to use all three activities interacting together, and Region 3 preferred the consultations and training.

Facilitating factors for maintenance of the intervention were mainly found in the inner setting of the organizations involved (Table 7). The social and financial support of the consultant’s manager and colleagues were considered as facilitating, as was the openness of other professionals to teamwork with disciplines other than their own. Other facilitating factors in maintenance were a mindset geared to a need for change within organizations and a mindset among professionals in social service organizations that not only focuses on social and psychosocial care but also pays attention to somatic care needs. Maintenance was also facilitated by concrete actions that helped prevent the drop-out of consultants, like sharing information with colleagues on the intervention activities performed. Also, use of the threefold consultation service was expected to be most sustainable over time when ownership is assigned to organizations in palliative care. Structural discussion of persons experiencing homelessness initiated by the palliative care consultant, e.g. once a month, could also contribute to sustainable, early, future-focused consultations. In the outer setting, a clear policy of financial support and clear regulations regarding indications concerning ageing and serious illness of persons experiencing homelessness would help in maintaining the intervention over time.

**Table 7:** Overview of all reported facilitators and barriers of the RE-AIM Maintenance dimension

CFIR domain	Facilitators	Barriers
Intervention characteristics	• Refining the intervention regarding availability of consultant, frequent evaluation of intervention activities, and MDMs as standard practice may contribute to sustained use over time (QM1.1)^a^ • Ownership of the intervention by organizations in palliative care in order to transfer palliative care knowledge to social service professionals (QM1.2) • Structural discussion of patients initiated by the palliative care consultant may contribute to sustainable, early, future-focused consultations (QM1.3)	• Unclear mutual responsibilities hamper sustained use over time due to complexity (QM1.4) • A shift in non-intervention-related tasks of the palliative care consultant could hamper collaboration as this could hamper availability and participation in intervention activities (QM1.5) • Small-scale and specific consultations may negatively affect maintenance and expansion of the intervention over time (QM1.6)
Outer setting	• A policy of clear incentives and regulations regarding patient indication and associated existing funding help in maintaining the intervention over time (QM1.7)	• Lack of clear policy and regulations regarding proper/structural palliative care indications and funding for care (QM1.8)
Inner setting	• Mental and financial support from manager and colleagues for consultants within the organization helps prioritize intervention for consultants in future (QM1.9) • Professionals’ openness to teamwork helps use of the intervention over time (QM1.10)• Organizations’ recognition of a need for change contributes to a culture that is more open to change over time (QM1.11)• A mindset within social services that focuses on more domains such as the somatic domain (QM1.12) • Concrete actions, like sharing information on the intervention activities performed, to prevent loss of consultants’ position, helps embed intervention over time (QM1.13)	• As long as organizations are unfamiliar with death and dying among this population, maintaining the intervention will be hampered by these assumptions. (QM1.14) • Staff shortages hamper use of the intervention over time (QM1.15) • Unpaid medical tasks not being taken seriously within organization may hamper the implementation climate in future (QM1.16) • Different views on ethical issues may hamper future collaboration (QM1.17) • Many layers of management in an organization mean it takes a long time to arrange financing and hours for consultants; this threatens continuation of the intervention (QM1.18) • Drop-out and vulnerability of consultants’ position threaten use of the intervention over time (QM1.19)
Process		• Maintenance is highly dependent on local champions, which might threaten future collaborations and maintenance of the intervention over time (QM1.20)

^a^QM refers to quotes on Maintenance by facilitating and hindering factor, shown in Additional file 2: Appendix 2

The factors mentioned as barriers for maintenance of the intervention were mainly in the inner setting of organizations too. Unfamiliarity within organizations with death in the population could hamper the willingness to continue the intervention. In addition, staff shortages within the social services could hamper further maintenance of the intervention. So could drop-out and the vulnerability of the consultants’ position due to the dependency on a one-person position. Finally, in the outer setting, a lack of clear policy, funding and regulations regarding care for seriously ill persons experiencing homelessness on the part of the government and health insurers was considered as a barrier.

## Discussion

Results were structured using the Reach, Adoption, Implementation and Maintenance dimension of the RE-AIM framework and corresponding facilitators and barriers. The persons experiencing homelessness that were reached by the intervention were mostly seriously ill persons experiencing homelessness in the last days of life residing in shelters. The reach of the intervention was mainly accomplished by involving social service professionals who were working in the organizations that initiated the intervention. While adoption of the three activities of the intervention was achieved in all regions, there were differences in the start, timing and frequency of the three activities in each region. Implementation of the intervention was partly accomplished according to plan. Half of the consultations were bedside consultations and half were telephone consultations instead of the planned bedsides consultations. Also, instead of the planned duos of consultants, two of the three regions had collaboration between a palliative care consultant and a team of social service professionals. Also, the consultations were mainly in one direction, with palliative care consultants advising social service professionals. Finally, regarding maintenance, all regions expected to use one or more activities of the intervention in the future, although they differed in which activities they expected to use. Facilitators and barriers were found for all the RE-AIM dimensions; the facilitators were mainly found in the inner setting of the organizations, in characteristics of individuals, and in intervention characteristics. Barriers were mainly identified in the inner setting of the organizations and in intervention characteristics.

### The three activities of the intervention are closely related

Our study shows that the consultations, MDMs and training are interrelated and that all three elements are important in improving palliative care. Moreover, the elements reinforce each other as by working together the professionals know more easily how to find each other and know how to formulate a request for advice. Our study also shows that participants get to know one another through the recurring meetings in person, such as on training sessions, at bedside consultations or in MDMs. Thus, training sessions and multidisciplinary meetings might be especially relevant when starting a similar intervention, as they nurture a collaboration in which consultations can then be requested. Other interventions aimed at collaborations between palliative care and social services for this population are still scarce. International literature on this topic is still scarce, however, two British studies evaluated one intervention focused on palliative care specialists training, supporting and advising shelter staff; they also found that training, structural connections and advice reinforce each other [30, 31].

### The threefold intervention takes time and effort to adopt

This process evaluation reveals that implementation of an intervention focusing on palliative care provision requires time to create awareness and break down resistance and lack of knowledge about palliative care and dying. Specific issues to tackle are awareness raising, skills in recognizing and discussing serious illness and the end of life, norms and values about palliative care; and support of managers. This need for creating awareness and break down resistance among professionals is also found in one other study [31]. Although this study indicates that time and effort is needed for adoption and implementation, our study on added value suggests that added value can already be achieved in this phase of adoption and implementation [27]. Further, this process evaluation showed that efforts must also be made in the financial field: multidisciplinary care also needs multidisciplinary, structural funding to achieve long-term improvements in the palliative care for this population. However, since both the population and interventions regarding palliative care are understudied yet, evidence-based models of improving palliative care hardly exist [16, 32]. We recommend further research evidence-based interventions and evaluating the processes.

### The challenge of connecting two worlds

Our findings show that the inner setting is a determining factor and potentially a barrier in the process of adopting, implementing and maintaining the intervention. The settings of social services and palliative care differ substantially, e.g. in attitudes towards death and dying, skills in this area, organizational structures and recognition of the relevance of palliative care. A gap between social services and palliative care services as well as unfamiliarity with palliative care in social services is also found in other studies studying persons experiencing homelessness [33, 34]. Moreover, studies into populations of persons with mental illness and persons with intellectual disabilities show similar challenges in lack of training expertise among professionals [35] and issues with understanding the patients’ perspectives, referrals and collaboration between professionals in different disciplines, and training professionals in providing palliative care [36–38]. Taken together, this implicates that a palliative care intervention could be best implemented within social service providers, and palliative care professionals should have a proactive role in the provision of consultations and training and participation in MDMs.

### Strengths and limitations

Our study is the first study to combine consultations, multidisciplinary meetings and training, with palliative care professionals and social service professionals in an intervention. Another strength of this study is the process evaluation using RE-AIM and CFIR sequentially, resulting in structured domains of facilitators and barriers. Also, both qualitative and quantitative data collection resulted in a broad scope covering different dimensions of RE-AIM. A strength of this study is that this intervention is based on the needs and wishes expressed by persons experiencing homelessness. They indicated that professionals needed more knowledge, training and collaboration in palliative care [19]. The design of this intervention and the evaluation of the intervention were supervised by an advisory board, in which people who experienced homeless were also represented. A limitation is that we did not interview persons experiencing homelessness who received palliative care; this study therefore gives the professionals’ views on their situation rather than their own perspective. Furthermore, a limitation is that we could only estimate which professionals could have benefited from the intervention in relation to which professionals were reached by the intervention. We could not compare characteristics of professionals who were not reached to professionals who were reached by the intervention In relation to this, we did not question professionals who did not use the intervention about why they did not, while they could have given more insight in barriers of implementation.

## Conclusions

A threefold consultation service aimed at improving palliative care provision to persons experiencing homelessness was implemented, with consultations, joint multidisciplinary meetings and training initiated by palliative care professionals. It proved possible to implement the intervention, especially when it is tailored to fit the specifics of the region and sufficient time for adoption and implementation was allowed. It is important to allowing variation to fit the context, such as doing both bedside consultations and telephone consultations or connecting a palliative care consultant to a team of social service professionals rather than to an individual social service professional. We recommend further implementing this region-tailored intervention within social service teams, with competent and enthusiastic palliative care consultants in the lead. The intervention can start with training to raise awareness of possible palliative care needs and reduce fear of palliative care provision among social service professionals.

## Supplementary Information

**Additional file 1. **Semi-structured group interviews with attendees of MDMs and training activities.

**Additional file 2. **Overview of barriers and facilitators, organized by RE-AIM elements and main CFIR domains.

## Data Availability

The datasets generated and/or analyzed during the study are not publicly available due to the small scale of this intervention and evaluation and the easily traceable nature of de data, but are available from the corresponding author on reasonable request.

## Abbreviations

GP: General Practicioner
MDM: Multidisciplinary meeting
CBS: Statistics Netherlands
RE-AIM: Framework to evaluate Reach, Effectiveness, Adoption, Implementation and Maintenance of an intervention
CFIR: Consolidated Framework for Implementation Research
COREQ: Consolidated Criteria for Reporting Qualitative Research
