# Supplementary material for: Implementation of a threefold intervention to improve palliative care for persons experiencing homelessness: a process evaluation using the RE-AIM framework
Source: BMC Palliat Care. 2022 Nov 4;21:192. doi: 10.1186/s12904-022-01083-3 (PMC9635139; doi:10.1186/s12904-022-01083-3)
Supplement: Supplementary file 1 — Additional file 1. Semi-structured group interviews with attendees of MDMs and training activities. [file 12904_2022_1083_MOESM1_ESM.docx]

Appendix 1. Overview of topic-list used for semi-structured (group) interviews

**Semi-structured group interviews with attendees of MDMs and training activities**

Process of organizing MDMs and training activities

- Start and progress
- Establishing contact and collaboration
- Role of consultant
- Appreciation of meetings attended

Perceived added value

- Of topics discussed
- Of MDM or training for professionals and patients
- For daily work and competences
- Contribution to identification of patients with palliative-care needs
- Contribution to quality and timing of palliative care

Maintenance and suggestions for improvement

- Advice for consultant/trainer
- Advice for other regions

**Semi-structured individual interviews with managers of the organizations involved**

Process of consultations, MDMs and training

- Start and progress
- Establishing contact and collaboration
- Facilitating the consultant
- Collaboration with other organizations

Perceived added value

- Of consultations, MDMs, and training activities for own organization
- Of consultations, MDMs, and training activities on palliative care for the homeless

Maintenance

- Future-proof financing of activities
- Ownership of activities

**Semi-structured individual interviews with the consultants involved**

Description of current activities

- Consultations, MDMs, training activities
- What works, what doesn’t?
- Collaboration with other professionals in palliative-care provision
  - What helps, what doesn’t?
  - Needs of care professionals
  - Strategies to involve other professionals

Implementing the intervention

- Minimal effort to let the intervention work
- Most useful elements of the intervention
- Missing elements

Added value and effectiveness

- Perceived achievements of the intervention
  - Through consultations, MDMs, training
  - On quality and timing of palliative care
  - On collaboration
  - On competences
