# Supplementary material for: Implementation of a threefold intervention to improve palliative care for persons experiencing homelessness: a process evaluation using the RE-AIM framework
Source: BMC Palliat Care. 2022 Nov 4;21:192. doi: 10.1186/s12904-022-01083-3 (PMC9635139; doi:10.1186/s12904-022-01083-3)
Supplement: Supplementary file 2 — Additional file 2. Overview of barriers and facilitators, organized by RE-AIM elements and main CFIR domains. [file 12904_2022_1083_MOESM2_ESM.docx]

**Overview of barriers and facilitators, organized by RE-AIM elements and main CFIR domains**

| **RE-AIM domain** | **CFIR domain** | **Facilitators** | **Barriers** |
| --- | --- | --- | --- |
| Adoption | 1. Intervention characteristics | Professionals perceive a need and priority for additional support which makes them feel the need for collaboration (QA1.1)  *“I think that has become clear, that I definitely have little to no expertise when it comes to the homeless — that is really her area of expertise. But I do have expertise when it comes to palliative care. So I think that makes it work. Right, you need one another for that, yes.” (Consultant 2)* | Benefit of consultations not recognized by all the professionals involved as not everyone feels the need to engage the consultant in bedside consultations (QA1.4) *“Right, I can imagine it could grow more into something where you go and look at the patient at some point. At the moment, it’s all advice on paper, as it were, because of course you only see the resident on paper. But there could come a point where that’s perhaps at the patient’s bedside.”* (Consultant 1) |
|  |  | Provision of palliative care tools helps consultants concretize palliative care in intervention activities (QA1.2)  *“Sent [consultant] tools because she needed them: alert line, advance care planning form, Utrecht symptom diary, palliative performance scale, Leiden conversation aid.” (Implementation logbook)* | Concerns about the relative advantage of the intervention as palliative care concerns a small population and narrow topic (QA1.5)  *“I think it’s the fact that the topic [palliative care] is quite limited in some way. It’s such a small thing. [...] It’s just difficult. You don’t see it happen much.” (Consultant 5)* |
|  |  | An intervention tailored to local collaborations and structures facilitates easy adoption of the intervention (QA1.3)  *“Right, look, we already had a setup, of course; we had that practice of course where [street doctor] is the practice holder, we have MDM discussions and that MDM for the vulnerable elderly, which we have once every six weeks or two months. So we already had a structure.” (Manager 2)* |  |
|  | 2. Outer setting | Pre-existing regular meetings in professionals’ network means that professionals who already have a network among other organizations can easily adopt the intervention (QA1.6)  *“Because... so we’d been together because of [city]. So both [hospice] and [other shelter organization] and [shelter organization] take part in project meetings and that kind of thing, as well as representatives from [other shelter organization] and [shelter organization] and [hospice]. So that means you already know one another and well, basically, you meet one another everywhere.” (Manager 3)* | Unclear policies on responsibilities. due to too many external parties involved in care for homeless people may hinder adoption of the intervention (QA1.7)  *“But I think precisely because of all the many options and disciplines in [city], if you have a specific question you often get lost in all the possible options. And sometimes you get shunted from pillar to post as a result. And I think that you... yes, that absolutely does happen, that people say that’s nothing to do with us, you should really go there. And then you go there and they say, well, no, we’re referring you back to... It is easier for people here in [city] to pass the buck to someone else again.” (Training 2)* |
|  | 3. Inner setting | Intervention is compatible within existing workflow due to a clear route of palliative care (and responsibilities) within the organization participating in the intervention (QA1.8)  *“Well, that we have an embedded roadmap for residents who have been given the diagnosis [final phase of life] or a pre-diagnosis, that you say that you don’t know. That you can take a proper roadmap off the shelf as a carer or nurse, and immediately start delivering the care. And it also specifies using a consultant. Embedding the MDMs, which are important. And she [the consultant] doesn’t always have to attend; you can say, well we have this resident, we’ll call on you. Because that care too changes somewhat for us.” (Manager 8)* | Norms and values within the organization for social service provision are often focused on social care, while somatic care gets less attention (QA1.11) *“That whole aspect is simply... not in this business... They find it scary too. And you see too that when the moment comes — because I think that’s also part of it, they don’t want to see it — because when the moment comes that you see someone is getting dirty for example, you should do something about it. It’s also often a kind of mantra: no, it’s physical, it’s to do with the body, and I’m not going to do anything about that because it would be bad for my relationship with my client. Whereas I think that doesn’t have to be the case at all. It could enhance it.” (Manager 1)* |
|  |  | Shared vision on good healthcare among colleagues within the participating organization makes collaboration easier (QA1.9)  *I know she was involved at [location 2] with someone who was really in the terminal stage... and also behaviourally... I’m not going to the nursing home. [...] She brings those different worlds together. You really need that with a specific group, especially here with ageing people and addiction and Lord alone knows what diseases. Yes, the whole package is broader, more complex perhaps. She’s better at that. I don’t have a nursing background at all. But the ultimate responsibility for the processes is mine, so I thought, ‘Oh great, someone who’s helping find solutions: that can only be positive.’” (MDM, U region)* | Limited skills in recognizing, discussing and providing palliative care within social services hinder adequate care at the end of life (QA1.12)  *“I certainly don’t have the knowledge about palliative care because if you can’t put into words what you see, someone’s deteriorating but you can’t put into words exactly what you see and you don’t know what action to take, or you can’t explain it to a GP, well I can understand why people don’t get noticed, or they get noticed far too late. [...] The problem is that sheltered housing supervisors don’t report... well, they report a deterioration but they can’t tell the GP exactly what it is they’re seeing. ‘The person’s a bit under the weather’. And right, that’s as much as you’re getting.” (Consultant 4)* |
|  |  | Shared views of involved professionals regarding equal and reciprocal cooperation (QA1.10) *“It’s on an equal footing because I always say, ‘I know a bit about palliative care and you know a bit about homeless people and that’s how we complement one another’. I can also say ‘I know an awful lot and you know an awful lot, but we complement one another’.” (Consultant 2)* | Many staff changes and insecure future prospects for organization make organizational commitment hard (QA1.13)  *“Because of all the changes in personnel and responsibilities, too. We really need to appoint a single person who can deal with it and take charge and who also makes sure it gets picked up in the team. Well, we did have someone but then our whole team got reorganized again. Then you have more new people so you really start from square one again at the start of this year.” (Manager 8)* |
|  |  |  | Limited support and engagement of management makes consultants feel they are getting little support (QA1.14)  *“Consultant feels manager is not giving much support or clarity, for example regarding time limits, available hours, a close colleague to work with, proper agreements, direct lines of communication.” (Implementation logbook)* |
|  | 4. Characteristics of individuals | Commitment and enthusiasm of the professionals involved regarding the intervention and palliative care (QA1.15)  *“He said, ‘That we get listened to and noticed, that we get to tell our story’. He even said something I found quite funny: ‘I’m getting enthusiastic about palliative care’.” (Manager 1)* | Low self-efficacy in palliative care skills may hinder identification of palliative care and use of the intervention (QA1.23) *“And if it [death or imminent death] happens, sometimes it feels as if it slips through your fingers, so you think: oh, it happened again and perhaps we should have paid it more attention.” (Consultant 5)* |
|  |  | Medical skills and knowledge of individual consultant makes professionals feel the consultant is competent (QA1.16)  *“And so I see in my partnership with [other consultant], they are genuinely saying hey, that person has even more of a medical perspective than me of course, my medical perspective has already become incredibly muddied by everything I’ve seen and she still sees someone’s somatic condition and think hey, that’s what’s going on.” (Consultant 5)* | Professionals’ differences in their views when a consultation is requested could hinder use of the intervention (QA1.24) *“Tricky when a colleague asks for advice in a situation where we don’t agree. The colleague doesn’t see it as a problem.” (Implementation logbook)* |
|  |  | An approachable consultant without their own agenda helps the professionals to adapt the intervention according to their needs (QA1.17)  *“Basically that you go round there with a fairly low profile. Of course I don’t really have any particular status; I don’t have to do anything. I think it becomes complex for them because, well, then they have to do something. [...] So if I take a serious stand there and say all kinds of things need to happen, then I think I would lose them.” (Consultant 1)* |  |
|  |  | Adoption is facilitated by consultants who proactively initiate consultations, organize training or participate in multidisciplinary meetings, which makes it easier for professionals to adapt to the involvement of the consultant (QA1.18)  *“Yes, that’s simply keeping in easy contact with the locations. [...] And if I visit the location myself, I also like to pop in on the most vulnerable people, the ones I’m really worried about, let’s say, who just aren’t doing well. So I drop in on them, after agreeing it with the sheltered housing supervisor. She’s always really pleased about that, or she comes along with me, right... Right, I find it goes very well like this. And I find that people really appreciate it. And if I phone them between times too, and ask how things are going, are there any new developments, can I help them with anything? Then I find that they really appreciate that a lot.” (Consultant 5)* |  |
|  |  | A consultant who is familiar with the homeless population makes the intervention easier to adopt for social professionals (QA1.19)  *“You also have doctors who say well, if he’s using cocaine... no, then you can’t do this... or you can’t do that. That isn’t an issue. That’s what makes it so difficult to explain what the problems are with our specific group in the regular health service. [...] She [consultant] does know that there’s no point in questioning that. Sure, you can say it’s not such a good idea for him to walk around outside drunk, that you should go and fetch him every time he’s outside, you know. Sure, everyone realizes this. But that situation’s not an issue for her.” (Consultant 3)* |  |
|  |  | Open mindset and attitude of professionals helps make them more motivated for palliative care (QA1.20)  *“But also really the open mindset of our nurses; nothing is a standard, a protocol. Because we know that it’s different for everybody. And I think we’re different to the regular nursing-home care, where you base things more on a protocol, and that’s just how you do that because you’ve been doing it that way for so long. Here you tend to take a tailored approach. And I think that helps a lot in that final stretch of care.” (Consultant 3)* |  |
|  |  | Awareness of skill shortcomings makes individuals open to reflecting and learning (QA1.21)  *“That too helps make you kind of... you find out: hey, perhaps I was unconsciously competent. That awareness has developed in me, there’s this unconscious competence that makes me think: hey, that’s also something other care providers have too to a certain degree for a particular case. It’s good to develop that awareness so that you can also communicate that: hey, you might have more know-how than you realize.” (Consultant 5)* |  |
|  |  | Individuals who trust each other cooperate better in using the intervention (QA1.22)  *“Right, I think a certain calm... you do see a lot of discontinuity in the care providers. And yes, trust in one another. Knowing that the team meetings have a really useful purpose at any rate. I think knowing one another a bit can definitely help. Sometimes you all kind of need to have a common enemy, shall we say.” (Manager 7)* |  |
|  | 5. Process |  | Issues with converting the work plan and intentions into actions impedes planning and engaging appropriate individuals (QA1.25)  *“It’s all still somewhat vague. Group members will think about their roles. Starting in September turns out to be too ambitious because of holidays and staff shortages.” (Implementation logbook)* |
|  |  |  | Unclear implementation route for intervention within organization (QA1.26)  *“More than you thought... Ah, so that’s what palliative care is. We had to learn that. We should have announced it differently beforehand, that’s why there were some delays sometimes. [...] She should have come along to the team meeting and explained publicly what we were going to do. And made a plan based on that. In the end, it was all a bit messy and people didn’t know exactly what she was supposed to be doing. I didn’t know either... are you going to bring her in for individual cases... I mean, who was in the terminal phase anyway, we thought? No one. We kind of went from Z to A instead of from A to Z.” (MDM, U region)* |
| Implementation | 1. Intervention characteristics | Frequent physical meetings (consultations, MDMs, training) normalize collaboration between palliative care professionals and social service professionals (QI1.1) *“And we have that now... Right, we see each other even more now, we are in contact even more often and so it’s easier to take action, let’s say.” (MDM, R Region)* | Unclear role of consultant negatively increases the perceived complexity of the intervention (QI1.6)  *“Well, at the beginning I remember it was a bit chaotic starting up the consultations; we didn’t really know what we were going to do exactly and when.” (MDM, U Region)*  Making limited use of bedside consultation possibilities prevents the consultant from making their own assessments as intended in the intervention (QI1.7)  *“Look, it’s always nice, and I say this deliberately, nice to see a patient. [...] [With a phone consultation] you are ever so aware that you are always dependent on the picture that the person requesting the consultation gives of the situation. So the challenge then is to ask the right questions so you can get as complete a picture as possible.” (Consultant 2)*  Still no perceived necessity for MDMs in which palliative care is embedded (QI1.8) *“I think the middle element is the hardest because you need to systematically free up time for it and then you have that problem that the supervisors are often not aware of the somatic demands, let’s say. So that’s why it’s really important that you’re at these team meetings; then you can also give input and turn lack of awareness into awareness. But it isn’t easy to get your voice heard in such a team meeting; people often don’t honestly see the need.” (Manager 1)*  Discussion of patients in MDMs is too short/limited, which hampers implementation of advice in MDM (QI1.9)  *“Then you discuss their caseload, let’s say. But of course those palliative patients can be in anyone’s caseload. Whereas if you discuss the palliative people specifically, then you would really need to have far more personal supervisors and nurses there because they all have a good picture of their own people.” (Consultant 1)*  Limited time for patient discussion in training hampers implementation of knowledge and skills in training (QI1.10)  *“Too much time was... of course it’s nice to know who’s in the group, but too much time was spent on that. So now an entire case was skipped simply because we had run out of time, and I found that a real pity.” (Training in region A)*  A follow-up consultation was not always possible (QI1.11)  “*The follow-up was tricky sometimes, but that was also familiar from the contact with [consultant on the homeless nursing ward], [...] but it’s difficult sometimes, just because of the work pressure, to find out in the short term whether the consultation or the advice worked or was suitable. I think that would complete your consultation properly, of course, if you do the follow-up too and hear whether it worked or whether you need to adjust the advice. That sometimes took a bit longer than you’d like.” (Consultant 2)* |
|  |  | Consultants’ structured questioning helps in implementing consultations (QI1.2)  *“She writes it up in this very staccato way, so you really get a kind of prescription from her. Like this is an important point, this is an important point, did you calibrate this? And she does this by... she really interrogates me, I give a sketch of the situation, I tell her what we’re doing and why we’re doing it this way. Then she asks more and more questions, how are you doing that, did you do it like that, did you think of that? Did the doctor think of that medication? Then she always produces this really clear, compact report... which can go straight into the file as a guide for the nursing interventions, but the doctor can also look at it too, and maybe think ‘OK, that medication could be an idea’, or ‘Oh, so that one isn’t working so well’.” (Consultant 3)* |  |
|  |  | The three intervention activities complement each other, making implementation of the intervention easier (QI1.3) *“So you see that those three pillars [of the intervention]... they influence one another. And that is because you really... there is nothing complicated about that with meetings or so, of course there are a few meetings that are linked, but it really happens automatically. She knows what I do; I know what she does. That other person experiences various things and knows what I do in the MDM group, which she goes to as well.” (Manager 2)* |  |
|  |  | Discussing patient cases in training helps participants to see the advantage of training (QI1.4)  *“Yes, I found it a nice set-up. First some general knowledge and then looking more at case studies together, plus we had a few who were also in the group and had personal experience and they could talk about this part or could ask questions, then finishing with case studies.” (Training in region A)* |  |
|  |  | Making notes of conversations and appointments in patient files contributes to clear agreements (QI1.5) *“And all the things I do during a peer-review session are recorded in the GP file. So [the street doctor] notes down the people who are discussed in the file.” (Manager 2)* |  |
|  |  |  |  |
|  |  |  |  |
|  | 2. Outer setting | Familiarity with other professionals (not participating in the intervention) through pre-existing regular meetings helps in implementing the intervention (QI1.12)  *“Well, we already had regular meetings and so on. So we were already discussing homeless people with complicated stories. And I think that has become even more solid, let’s say.” (MDM, R region)* |  |
|  |  | A policy of clear incentives and regulations regarding palliative care indication and associated (existing) funding helps in implementing the intervention (QI1.153) *“Yes, that’s the mental healthcare under the Long-Term Care Act, that applies to these patients too, and that means... we’re still figuring out... because it includes some personal care, so should that be delivered at the location where they live? That’s a new aspect. Until recently, it was like, ‘Oh no, this is to do with the body so that’s someone else’s business, something for the nursing staff.’ Now you see a tendency to say, ‘Hey, we should be organizing this ourselves’ – but how are we going to do that actually?” (Manager 1)* |  |
|  | 3. Inner setting | Available time of consultant supports adaptability of the intervention (QI1.14) *“Physical space and time, plus the prerequisites (a PC and phone), of course. But that’s a bit basic — banal, I would almost say. But I’m the right person in that case, because I really am in charge of my own work.” (Consultant 2)* | Staff shortages hinder implementation the intervention (QI1.15)  *“Because if you don’t free up someone and you tell them ‘Do it on the side’ or whatever, then it just doesn’t work. The routes are always full, there is never any time. [...] And people get completely taken up with the day-to-day business. So it’s a good idea to schedule some free time for people, otherwise it doesn’t work.” (Manager 1)* |
|  |  |  | Many unexpected events and therefore ad hoc activities within social service organizations distracts from focusing on implementing the intervention (QI1.16)  *“Indeed, that aspect of yes, we need to look at this, we shouldn’t skip it because it’s already so hectic and busy. Right, pay a bit more attention to it. Right, and everyone does do that. The nurses working here all have their hearts in the right place. But it gets incredibly busy sometimes and then you do sometimes overlook things.” (Manager 6)*  Implementation can be difficult when social service professionals do not know the consultant yet (QI1.17) *“In December last year, we had an exploratory meeting for the first time about caring for the carers. That was very exploratory. That may have been disappointing for [consultant on the homeless nursing ward], but you can’t do a peer intervention on caring for the carers straight off — first you need to know one another and know what you are dealing with.” (Consultant 2)* |
|  |  |  | Lack of knowledge on how to recognize palliative care needs frequently led to consultations that were too late in the illness trajectory (QI1.18)  *“Consultant was brought in for a palliative case. He was brought in at a late stage: will the patient survive to the weekend?” (Implementation logbook)* |
|  | 4. Characteristics of individuals | Consultant is experienced in advising professionals about this patient population, which helps in introducing implementation activities (QI1.19)  *“She is so at home with these kinds of problems that we can get a lot of help every time. You think to yourself that doctor knows it all, he knows what medication he should prescribe but he can still get ideas because she asks specifically about something, ‘OK, nauseous and so on, well, did you think of that? Have they already tried that?’ Terminal care, that final care delivery, is such a specialist field, you know, that you think, ‘Oh yes, of course, yes, of course’ when she says all this. Then I think, OK, of course. It’s not a matter of course for me because it’s something you do in addition to your own work.” (Consultant 3)*  Predictability on consultants’ presence helps to normalize implementation of intervention activities (QI1.20)  *“But what I’ve had in the past is, ‘Oh, [consultant] is here this week, how nice.’ So we weren’t always particularly switched on to this. And of course that’s because that person’s there the one time, but not the next time. And sometimes she would have a course the next time so she would skip that time again. So there was a bit less continuity in that respect, for example. But fortunately [consultant] keeps that continuity up to scratch.” (Manager 5)* | Resistance and fear of end of life and death hinders implementation (QI1.25)  *“Consultant noticed resistance in the team: we have nothing to do with palliative care, why are we looking at this, we don’t have that here (focused on short-term stays). In the course of the discussion, recognition that this [dying] did indeed happen, signals.” (Implementation logbook)* |
|  |  | Sharing tasks with a colleague helps familiarization with implementing the intervention (QI1.21)  *P: “And if someone has taken that step [to deliver palliative care], then you’ll need more time for that because you need to schedule those consultations, you’ll want the geriatric specialist to have a look too, and there are other things that are needed too.”*  *I: “Right, so do I understand correctly that you simply do it in your normal working hours?”*  *P: “Yes, I can easily manage that. That’s because there’s two of us. So if I have to spend time on something, it takes priority for the moment and my colleague simply does whatever else it is that needs doing.” (Consultant 3)* |  |
|  |  | Getting on as consultants helps in implementing consultations (QI1.22)  *I: “What works well in these consultations, between you two as professionals?”* *P: “Well, to be very subjective, we like one another. It’s accessible.” (Consultant 2)* |  |
|  |  | A personal bond between social service provider and patient helps in ensuring proper use of the intervention (QI1.23)  *“But I do think that having the supervisors involved makes a really big difference in what a patient wants or doesn’t want [regarding care] in the end. [...] What I see happening at the locations is incredibly positive. It makes me think: right, the supervisors have built up so much, when I see their dedication... Right, personal supervisors who have various patients and know absolutely everything about them and want the best for them and do their utmost, and also regularly have a bit of a one-to-one chat. The people there are incredibly good at the sheltered housing supervision side.” (Consultant 4)* |  |
|  |  | Professionals perceiving the consultant as highly competent (QI1.24) *“She [consultant] knows this target group. Our psychiatrist was a GP so that person also knows a lot. But they’re basically a normal GP, so not part of the [organization]. And you see that [consultant] has experience with people with multiple diagnoses, so the addiction, the care avoidance, the smoking, the behaviour extremes that you don’t come across that often as a normal GP. And she is better at making that link, which is what I hear from other people. [...] She knows the specific group very well and she knows about the medical side. That is indeed the area where she knows a bit more than what we know as sheltered housing supervisors.” (MDM, U region)* |  |
|  | 5. Process |  | COVID-19 restrictions and the scaling down of healthcare made planning and implementing intervention activities more difficult (QI1.25) |
|  |  |  | *“Right, and then there was Covid. We had a plan for training, with various peer group intervention sessions for care for the carers, then in addition to that training in palliative care, mainly pharmacological but also sedation, for example. That’s still the intention.” (Consultant 2)*   *“But I’m waiting a bit with that [organizing MDM] in the hope that the coronavirus crisis lets up a bit. Because that’s the tricky thing now: we always had a team meeting where we had invited speakers, and of course that lets you tell the whole team about things in one go in a nice, interactive way. But at the moment we’re only allowed a maximum of ten people at a meeting. And that wouldn’t even get me half my team.” (Manager 5)*  *“And right, I paid what I’d call a home visit on one occasion, so where I went to see the patient with [consultant on the homeless nursing ward] and I saw them. Of course that’s become less now because of Covid. [...] I don’t remember so well, I think it was the end of last year. This year, the consultations have mainly been by phone.” (Consultant 2)* |
| Maintenance | 1. Intervention characteristics | Refining the intervention regarding availability of consultant, frequent evaluation of intervention activities, and MDMs as standard practice may contribute to sustained use over time (QM1.1)  *“According to the GP involved, prerequisites for palliative care delivery at a location where homeless people live are: an [advisory] team that is prepared to be available during evenings.” (Implementation logbook)*  *I: “What do you need to do to make sure it [the intervention] can take place?”*  *P: “Well, to start with, have a chat soon with [consultant] to evaluate whether this use of her valuable time is something that she also sees as worthwhile, let’s say. Also, towards the end of the year, when looking at the plan for 2021 and the training policy, have a look to see whether there are colleagues who could benefit from this training or that [palliative care] course.” (Manager 5)*  *“Right, what I’d like to work towards is not just having [social care consultant] responsible for this but getting the whole team involved. So in my ideal world you have an MDM with more people than just [social care consultant]. [...] Well, in my previous job I was used to sitting down with the people involved around the table. So with a fixed MDM appointment where the duty nurse joins in and our social worker can join in too. That gives you the full picture. And you get a broader support base. Then you don’t have to hand it over, that’s not always what works.” (Manager 6)* | Unclear mutual responsibilities hamper sustained use over time due to complexity (QM1.4)  *“What I discovered last week is that there are collaborative agreements between [employer] and [nursing ward for homeless people] that I’m fairly sure I didn’t know about and nor did [geriatric specialist, on call for palliative care consultation]. [...] I said I would really like to know what those collaborative agreements are because then you could see whether you could pay a home visit based on those agreements, let’s say. You know... But that’s being looked into... We need to look into that because it could easily be for home help, to give a crazy example. That’s not actually the case, of course, but as an example. So I need to look into that.” (Consultant 2)*  A shift in non-intervention related tasks of the palliative care consultant could hamper collaboration as this could hamper availability and participation in intervention activities (QM1.5) (Manager 1) |
|  |  | Ownership of the intervention by organizations in palliative care in order to transfer palliative care knowledge to social service professionals (QM1.2)  (Manager 1)  Structural discussion of patients initiated by the palliative care consultant may contribute to sustainable, early, future-focused consultations (QM1.3)  *“We are still doing it too much on an* ad hoc *basis, but perhaps it should be more preventive and anticipatory, so you make it even more of a useful instrument by saying we need to have a regular MDM once a month, just for the people on [homeless nursing ward]. Then everyone gets together and all the residents get discussed at the MDM.” (Manager 2)* | Small-scale and specific consultations may negatively affect maintenance and expansion of the intervention over time (QM1.6)  *“Heard from both [region 2] and [region 3]: Is it actually possible to offer 24-hour consultations when you have a small-scale duo consultation function? How can we set up the availability?” (Implementation logbook)* |
|  | 2. Outer setting | A policy of clear incentives and regulations regarding patient indication and associated existing funding help in maintaining the intervention over time (QM1.7)  *“Look, the best thing of course would be for the consultation function to be properly embedded in your... in your indication, so that you can simply make use of it and can have it as an indication, like community nursing. I think the most important thing is to have some kind of performance scheme with the insurers, in the future as well for all palliative care.” (Manager 8)* | Lack of clear policy and regulations regarding proper/structural palliative care indications and funding for care (QM1.8)  *“Even all the divisions, whether or not there are nursing beds there... of course that’s... right, the insurer wanted to make huge cuts in our funding because they said we don’t have so many primary-care beds for the homeless in [another city]. And if that’s going to be the model, that we aren’t allowed to put people in primary-care beds because then they have to be paid through the social care funds, whereas they are simply lying there in the palliative stage, as it were.” (Manager 7)* |
|  | 3. Inner setting | Mental and financial support from manager and colleagues for consultants within the organization helps prioritize intervention for consultants in future (QM1.9)  *“Well, look, what I could do is encourage her manager more so she frees up time for this. [...] But it’s also more about giving support, prioritizing, that she needs more support in this from her manager.” (Manager 8)* | As long as organizations are unfamiliar with death and dying among this population, maintaining the intervention will be hampered by these assumptions (QM1.14) “*They find death and so on – people dying – that’s all a bit scary. It’s like putting people in the shower: they even find that scary. So someone who’s dying — well, of course that’s quite stressful. And we have [shelter organization] for this. Look, perhaps it will be needed more in the future, but I’ve really noticed in the past that it’s not yet... that it’s a difficult topic.” (Manager 4)* |
|  |  | Professionals’ openness to teamwork helps use of the intervention over time (QM1.10)  *“Well, I think so after a year... I reckon everyone finds it ever so important to have [consultant] there and have her giving her input, yes, I would definitely say so. [...] Well, one example I can give of this is that [consultant] gets a phone call to tell her the MDM has been cancelled, let’s say. If you really don’t feel there’s any kind of benefit to having [consultant] there, or if you’re hardly aware of any benefit, then she wouldn’t get a phone call about it being cancelled.”* *(Manager 3)* | Staff shortages hamper use of the intervention over time (QM1.15)  *“If it [the intervention] were used properly, I think we wouldn’t have enough staff. Because I believe it [palliative care] is needed an awful lot but simply isn’t given. I honestly believe that.” (Manager 1)* |
|  |  | Organizations’ recognition of a need for change contributes to a culture that is more open to change over time (QM1.11) *“Because it seems there is... and that’s how things work in [care organization]... there’s suddenly a realization at the top that hey, something’s going on, and that can lead to a change in the culture. Because our specific group is becoming sicker and sicker, older and older, and it’s increasingly necessary. So I do see the organization doing something about this.” (Consultant 5)*  A mindset within social services that focuses on more domains such as the somatic domain (QM1.12) (Manager 2) | Unpaid medical tasks not being taken seriously within organization may hamper the implementation climate in future (QM1.16) “*Well, I think that as managers at [organization with homeless shelter], we need to agree amongst ourselves much more that we should simply be paid for the work we do. That has really turned into a kind of chronic discussion, that we don’t get paid for everything we do. [...] That’s partly to do with our image: that you stick plasters on but it’s not much more than that.” (Manager 1)* |
|  |  | Concrete actions, like sharing information on the intervention activities performed, to prevent loss of consultants’ position, helps embed intervention over time (QM1.13)  “They [consultant and manager] don’t see drop-out as a major problem because the consultant makes notes of the discussions she has, so her knowledge can easily be handed over.” *(Implementation logbook)* | Different views on ethical issues may hamper future collaboration (QM1.17)  *“But what we had occasionally in the period when we were working a bit more intensively with [hospice]... [...] We also had some patients who had active wishes during the palliative process they were in to have euthanasia — what’s the term for it? — applied. Right, and [hospice] didn’t go along with that. So on the one hand that’s a gap. [...] So I can... who am I to judge the wishes of someone who feels their suffering is unbearable? And for us not to go along with that and not use that medication that I’m pretty sure is allowed by law in the Netherlands. So we need to think of a different option there if it’s not possible via [hospice].” (Manager 5)*  Many layers of management in an organization mean it takes a long time to arrange financing and hours for consultants; this threatens continuation of the intervention (QM1.18) (Manager 4) |
|  |  |  | Drop-out and vulnerability of consultants’ position threaten use of the intervention over time (QM1.19)  *“Right, of course it’s a problem that it depends so much on certain people. If [social care consultant] drops out for a while, that really doesn’t help matters.” (Manager 7)* |
|  | 4. Process |  | Maintenance is highly dependent on local champions, which might threaten future collaborations and maintenance of the intervention over time (QM1.20)  *“That’s right, you really rely on your network and on people being known. And that’s a vulnerability: if a colleague leaves who people know well, you’re left starting from scratch again. The infrastructure isn’t yet up to it, let’s say. [...] Perhaps new things will be developed again gradually, I don’t really know. But I have this idea, this feeling that perhaps there’s some credit to be gained there.” (Manager 1)* |
